# Neuroblastoma presenting like a Wilms’ tumor with thrombus in inferior vena cava and pulmonary metastases: a case series

**DOI:** 10.1186/2193-1801-3-351

**Published:** 2014-07-10

**Authors:** Genevieve Gaetan, Alain Ouimet, Chantale Lapierre, Pierre Teira, Herve Sartelet

**Affiliations:** Surgery Department, Hôpital Sainte Justine, Montréal, Canada; Medical Imaging Department, Hôpital Sainte Justine, Montréal, Canada; Paediatric Department, Hôpital Sainte Justine, Montréal, Canada; Pathology Department, Hôpital Sainte Justine, Montréal, Canada; Department of Pathology, Centre Hospitalier Universitaire Robert Debre, 48 Boulevard Serurier, Paris, France

**Keywords:** Neuroblastoma, Wilms’ tumor, Thrombus in inferior vena cava, Pulmonary metastases

## Abstract

Neuroblastomas and Wilms’ tumors are frequent pediatric solid tumors. The first is frequently detected in the adrenal gland and the second develops in the kidneys. The extension through the vena cava and the lung metastases are frequent in Wilms’ tumors and are rarely seen in neuroblastoma. We present the cases of three children with abdominal tumors with thrombus in the inferior vena cava and pulmonary metastases demonstrating a stage 4 neuroblastoma. The three male patients were between 23 to 48 months old. They presented an abdominal mass, near the superior pole of the kidney. Thrombus of the vena cava was showed on imaging studies in all cases and pulmonary metastases were always found. Catecholamine metabolites were present in the first case and negative in the two others. Two out of three patients had a radical nephrectomy. The pathological analysis always found a poorly differentiated or undifferentiated neuroblastoma without MYCN amplification and confirmed the vein tumoral thrombus in the second case. The evolution of the first two patients was unfavorable and the third is alive. Invasion of the inferior vena cava and pulmonary metastases in children with neuroblastoma is uncommon and can modify the surgical management.

## Introduction

The primary diagnostic considerations of a large palpable right or left upper quadrant mass in an infant or young child include neuroblastoma and Wilms tumor. Commonly, the evaluation of a child with an abdominal mass includes imaging and biological analysis to aid in the diagnosis of possible neoplastic disease and to facilitate the distinction between the two tumors. In rare cases, the diagnosis is not so simple. Many Wilms tumors present as large abdominal masses with invasion of the renal vein and extension into the inferior vena cava, and many clinicians consider the finding of tumor thrombus in the inferior vena cava suggestive of Wilms tumor (Bagatell et al. 2002) while it is extremely rare with neuroblastomas. Moreover, the most common sites of metastasis in neuroblastoma are the bone and bone marrow, with involvement of these sites found in the majority of children with newly diagnosed metastatic neuroblastoma. In contrast, lung metastasis is a distinctly uncommon finding in children presenting with metastatic neuroblastoma and remains a useful prognostic marker of unfavorable outcome (Dubois et al. 2008) while lung metastases are frequent in Wilms tumor.

In this context, we hereby present three cases of neuroblastoma in which a thrombus was found in the inferior vena cava associated with lung metastases and how it affected the clinical management and the prognosis of patient.

## Case reports

### Patient 1

**Figure 1:**
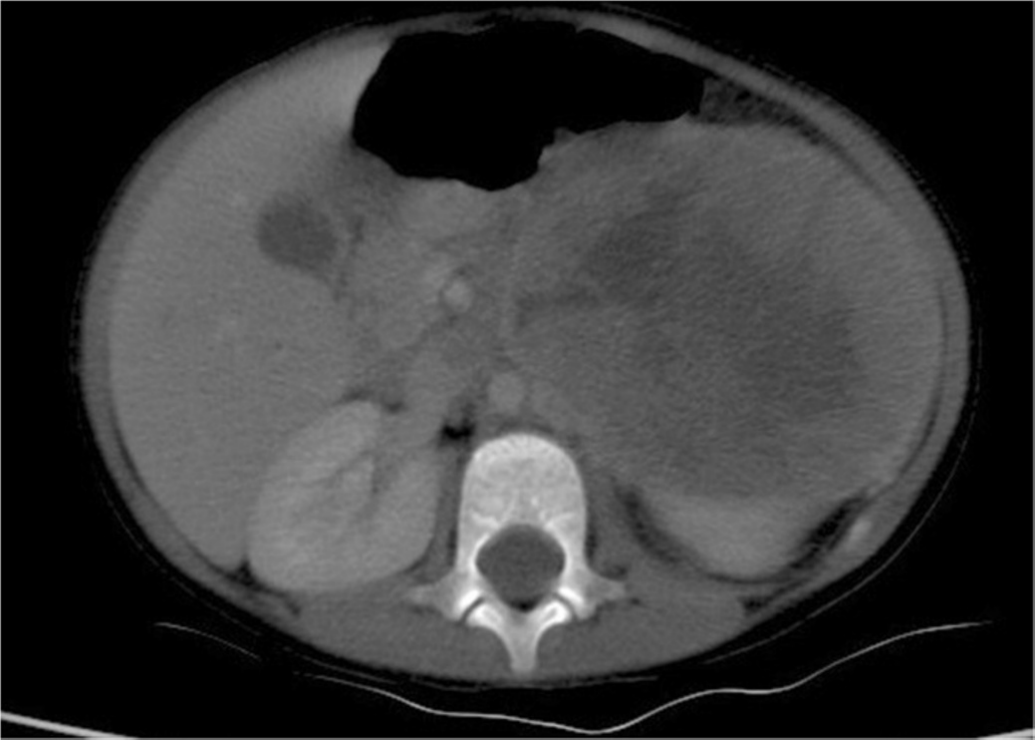
**Patient 1: Magnetic resonance imaging shows a large heterogeneous mass arising from the left adrenal gland associated with heterogeneous enhancement of the tumor thrombus in the inferior vena cava (arrow).**

The boy without personal or familial background had been suffering from fatigue for about a month associated with fever, at that age of twenty three months. Blood tests were performed, putting in evidence a normochromic anaemia, which justified his transfer to hospital. Upon his arrival, the boy was described as being pale, with tachycardia and a fluctuating temperature of a maximum of 39 C associated with hepatomegaly and splenomegaly. It was noted that the boy had lost more than one kilogram in one month. The blood tests demonstrated an important elevation of alkaline phosphatase and LDH levels. A VMA spot was positive. A chest x-ray showed an elevation of the left diaphragm. An abdominal ultrasonography demonstrated a large left retroperitoneal mass with necrosis and calcification, compressing the vessels and organs. An abdominal CT-scan and MRI were performed: the size of the mass was 88 × 127 × 140 mm, with solid and necrotic components (Figure 1). A thoracic CT-scan demonstrated multiple peripheral nodules, the largest being of 12 mm. An MIBG scintigraphy showed an intense uptake at the levels of the abdominal tumor, orbit, clavicle, femur and iliac bones. VMA and HVA levels were highly elevated, strongly pointing towards a neuroblastoma; a biopsy was made and showed a poorly differentiated neuroblastoma, intermediate MKI with unfavorable histology, and without *MYCN* amplification. The bone marrow biopsy was normal but bone marrow aspirate cytology contained metastatic tumor cells. Chemotherapy was therefore started, consisting of vincristin, doxorubicin and cyclophosphamide. The patient received a total of six cycles of treatment. Two months after the initial investigation, the CT-scan showed a progression of the tumour which now invaded the renal vein, mesenteric vein and celiac axis as well as the vena cava (Figure 1). Chemotherapy was continued for five cycles. One month after the fifth cycle of chemotherapy, the patient was brought at the hospital for deterioration of his general condition. A cerebral and orbital scan showed cerebral metastases, as well as a progression of the orbital lesion, extending in the skull. The child died in the following month.

### Patient 2

**Figure 2:**
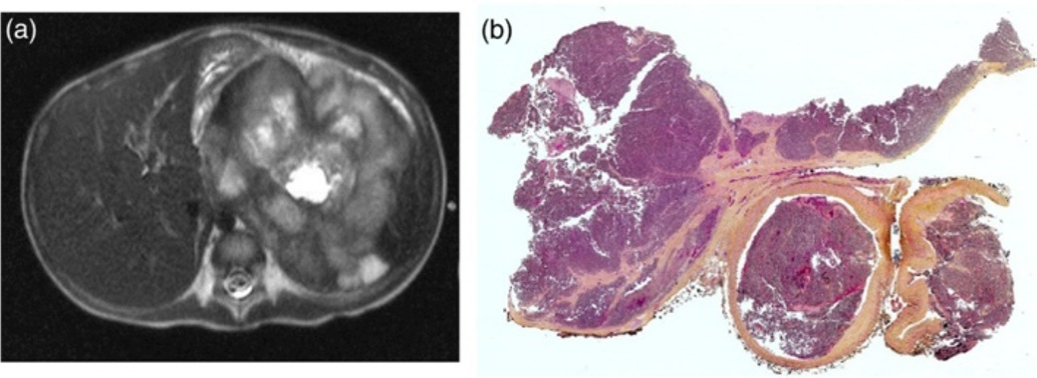
**Radiological and pathological analysis of the tumor of the patient 2. (a) Abdominal CT-scan**: large heterogeneous left side mass with a large tumor thrombus in the inferior vena cava (arrow). **(b)**: **histology of the renal vein**: venous tumor thrombus (25x magnification).

The patient was a boy who presented at four years of age with fever, diffuse abdominal pain, vomiting and loss of appetite. He was admitted to hospital where blood tests showed anaemia with neutropenia. The chest x-ray revealed an important pleural effusion, and an abdominal ultrasonography demonstrated a left 9 × 7.4 × 8.3 cm solid, well vascularized mass in the upper left quadrant. The physical examination done at that time described a palpable and painful mass in the upper left quadrant. An abdominal CT-scan showing a mass originating from the superior aspect of the left kidney, encapsulated, of 8.2 × 8.7 × 8 cm of size. Its centre was necrotic without calcifications (Figure 2a). Although the thoracic CT-scan demonstrated multiple pulmonary nodules; pleural drainage was done and discontinued after regression of the pleural effusion. HVA, VMA and Dopamine values were in the normal range. The presumed diagnostic was a Wilms tumour. In this context, bone marrow biopsy was not realized. Consequently, surgery for total left nephrectomy by laparotomy was scheduled. The tumour was adherent to the diaphragm and spleen. When ligating the renal vein, important hemodynamic instability ensued and was associated with pulmonary embolism, causing an estimated thirty minutes of hypoperfusion, requiring aggressive cardiac reanimation. A thrombectomy of the right and left pulmonary arteries was done under extra-corporeal circulation (ECC). The abdominal surgery was resumed and completed with partial resection of the diaphragm, splenectomy and hemostasis secondary to bleeding after use of ECC. The histological analysis of the tumor demonstrated the presence of a poorly differentiated neuroblastoma with high MKI with unfavorable histology, and invasion of the renal vein (Figure 2b) and pulmonary metastases. In primary location, the tumor was limited by a pseudo-capsule without infiltration of the adjacent kidney. *MYCN* was not amplified. Chemotherapy was started three weeks post-operatively and included vincristin, doxorubicin and cyclophosphamide, intercalated with treatments of cisplastin and VP16, followed by autologous bone marrow transplant (ABMT). Eight months after his initial visit, the patient was admitted for severe cephalalgia with vomiting. A cerebral scan showed extensive metastases with important mass effect. The clinical state went on deteriorating with evidence of brain herniation and focal neurologic signs. The patient died two days after his admission.

### Patient 3

**Figure 3:**
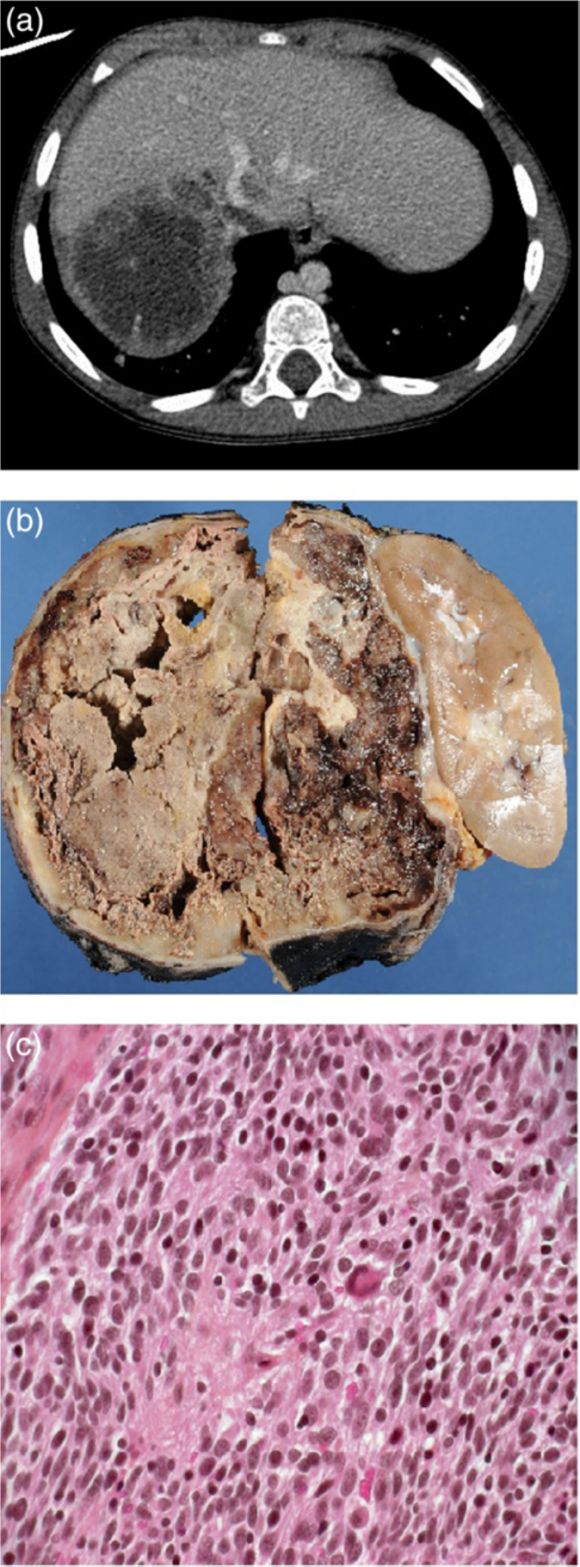
**Radiological and pathological analysis of the tumor of the patient 3. (a) Abdominal CT-scan**: large heterogeneous right abdominal mass with a tumor thrombus of the inferior vena (Arrow). **(b)**: **Gross examination of the tumor**: heterogeneous partially necrotizing tumor of the right adrenal gland. **(c)**: **Histology of the tumor**: neuroblastoma poorly differentiated (400x magnification).

A boy presented to his local physician for fatigue at the age of forty eight months. The physical examination revealed an abdominal mass for which the boy was sent to hospital. At arrival, the physical examination showed an easily palpable mass in the right abdomen extending from the costal margin to below the umbilicus and crossing the mid-line. The neurological examination was normal. Blood tests demonstrated an anemia with normal values for HVA-VMA. An abdominal ultrasonography showed a right renal mass with tumour in the right renal vein with extension to the inferior vena cava which was confirmed on the CT-scan (Figure 3a). Bone marrow biopsies were normal. Complementary work-up included a thoracic CT-scan showing pulmonary metastases. An MIBG scintigraphy showed low uptake in lungs predominantly on the right but no uptake on the tumor. The working diagnostic was that of a Wilms tumour. A right radical nephrectomy, taking care not to disrupt the venous thrombus before excision, was performed with a rapid recovery (Figure 3b). The pathologic examination demonstrated the presence of a poorly differentiated neuroblastoma and intermediate MKI (Figure 3c) and unfavorable histology. The tumor was well limited without infiltration of the kidney. *MYCN* was not amplified. The chemotherapy was started just after the histological diagnosis (6 cycles of cyclophosphamide, etoposide, topotecan, cisplatin, doxorubicine, vincristine) followed by intensification therapy with autologous bone marrow transplantation, abdominal radiotherapy and immunotherapy. Twenty one months after the end of the treatment and forty two months after the diagnosis, the patient is actually in complete remission.

## Discussion

Wilms tumor and neuroblastoma are the two most common abdominal neoplasms in young children. Both tumors could be localized in the upper abdominal quadrant and to discriminate between them could be difficult. It is essential to establish the correct diagnosis before surgery because it will change the surgical management. With a neuroblastoma, the surgeon will then determine during surgery if it is possible to separate or not the tumor of the adjacent kidney because tumors with thrombus in vena cava and pulmonary metastasis could directly invade the kidney.

However, each disease has specific characteristic features that may suggest a working diagnosis to guide evaluation and the initial surgical approach before histologic tissue analysis. The first clinical difference between the two tumors is the age at diagnosis. The peak incidence of neuroblastoma is at age 19 months and the distribution of cases by age clearly shows that this is a disease of infancy and early childhood (Brodeur et al. 1988) while nephroblastoma are more frequently seen in children older than age 40 months. In neuroblastomas with thrombus in the vena cava, the age at diagnosis of the tumor is always older than in classical neuroblastoma (Bagatell et al. 2002; Huang et al. 2006; Day et al. 1991; Mehta and Lim-Dunham 2003; Nagda et al. 2005) as for the patient in the present cases not allowing the distinction between neuroblastoma and Wilms tumor. Moreover, some cases have been discovered in adults (Custodio et al. 1999; Onishi et al. 1988; Yashiro et al. 1984).

Imaging exploration of abdominal mass in children often indicates the diagnosis in these tumors, but as these patients show, imaging studies may be misleading. For nephroblastoma we have the detection of the renal origin of the tumor with the claw sign but sometime this sign missing. The calcifications are more frequent in neuroblastoma but calcifications are also seen in approximately 15% of Wilms (Bagatell et al. 2002).

Concerning tumoral extension, the prevalence of tumor thrombi in the inferior vena cava for Wilms tumors has been seen in 4% to 19% of cases. Unlike Wilms tumor, this presentation is exceptional in pediatric neuroblastoma with only few case reports published (Bagatell et al. 2002; Huang et al. 2006; Day et al. 1991; Mehta and Lim-Dunham 2003; Nagda et al. 2005). Also in presence of an abdominal tumor with thrombus in the vena cava, the diagnosis of Wilms tumor is systematically evoked as in our last two cases.

Hematogenous metastases in Wilms’ tumor frequently involve the lung, liver and, rarely, bone, bone marrow, or brain while those of neuroblastoma are frequent in bone, bone marrow, liver and rare in lung. However, nearly 4% of patients with neuroblastoma presented lung metastases at diagnosis which was associated unfavorable outcome (Dubois et al. 2008). In our three cases, thrombi of the inferior vena cava are always associated with lung metastases which was confirmed by histology in the second case.

However, before surgery, specific additional analyzes are often used to differentiate neuroblastoma and Wilms’ tumor. Measurement of metabolites of catecholamine (VMA, HVA and dopamine) in urine is one of the most sensitive methods to diagnose neuroblastoma. More than 90% of the neuroblastomas are positive for one of the three parameters (Strenger et al. 2007) but also some tumors are negative as in our last two cases. In the same way, if neuroblastoma is suspected, the meta-iodobenzylguanidine MIBG scintigraphy is very specific to this tumor but about 10% of neuroblastoma do not present positivity. Also, non-secretory and MIBG negative tumors comprise 5% of all neuroblastic tumors. Also, in our series, the realization of MIBG scintigraphy could help establish the diagnosis of neuroblastoma and should be recommended in case of doubt about the origin of an abdominal tumor in children. An invasive exploration of the tumor by biopsy could also be discussed. However, a biopsy of a Wilms tumor before surgery may increase the stage of the tumor and can not be recommended in this situation.

The histological analysis of the neuroblastoma with thrombus in inferior vena cava revealed an aggressive tumor poorly or undifferentiated with intermediate or high MKI and unfavorable histology (Mehta and Lim-Dunham 2003; Nagda et al. 2005; Custodio et al. 1999). The neuroblastoma with tumor thrombus of the vena cava are highly aggressive tumors with bad prognosis and must receive a very intensive treatment to increase the possibility of a complete remission as in our last patient.

In conclusion, these three cases illustrate the importance of complete staging of an abdominal mass in view of treatment. If these cases are very infrequent, they typically demonstrated that in patient with invasion of the inferior vena cava and renal vein associated with pulmonary metastases, neuroblastoma must be eliminated even if it was a non-secretory tumor, in particular if surgery is considered. Not only does it complicate an intervention, it also dictates the prognosis and following treatment.

## Consent

Written informed consent was obtained from the patient for publication of this case report and accompanying images. A copy of the written consent is available for review by the Editor-in-Chief of this journal.
